# From remote screening to precision prevention: responsible multimodal AI for risk prediction and equitable oral healthcare

**DOI:** 10.3389/froh.2026.1878692

**Published:** 2026-07-24

**Authors:** Heydi Daniela Iglesias-Pérez, Maria Emilia Gallo-Sánchez, Ariel Sebastián López-Loachamin, Daniela Alejandra Paccha-Melgar, Julio Damián Rivadeneira-Ahuilar, Natalia Ibeth Luna-Ponce, Michael Alexis Gallo-Achig, Dayanna Michelle Pillajo-Villalba, Martín Campuzano-Donoso, Claudia Reytor-González

**Affiliations:** 1 Facultad de Ciencias Médicas, de la Salud y de la Vida, Escuela de Odontología, Universidad Internacional del Ecuador, Quito, Ecuador; 2 Center for Evidence Ecosystems, Implementation Science, and Decision-Making (CIDES), Facultad de Ciencias de la Salud y Bienestar Humano, Universidad Tecnológica Indoamérica, Ambato, Ecuador

**Keywords:** algorithmic bias, artificial intelligence, multimodal AI, oral cancer, oral health screening, periodontal disease, precision prevention, teledentistry

## Abstract

Artificial intelligence (AI) applications in oral healthcare have expanded considerably over the past decade. Deep-learning systems now report diagnostic performance exceeding 0.85 sensitivity and 0.90 specificity for several image-based tasks, with the highest pooled estimates reported in AI-assisted clinical photography for oral cancer and oral potentially malignant disorder (OPMD) detection (diagnostic odds ratio 68.4; AUC 0.938). These figures, however, mask three translational gaps. First, many studies often carry high risk of bias, lack external validation, and rest on heterogeneous reference standards. Second, datasets often provide limited demographic reporting, leaving uncertainty about model performance in the populations most likely to benefit from remote screening. Third, teledentistry and mHealth tools have outpaced the regulatory, validation, and fairness-auditing, and clinical-integration frameworks required for safe deployment. Preventive value emerges most clearly when AI is embedded in multimodal systems, such as imaging, sensors, behavioural feedback, clinician support rather than evaluated as an isolated classifier. Progress will be defined less by additional accuracy gains and more by external validation in diverse populations, transparent demographic reporting, equity-focused evaluation, and integration into prevention-oriented care pathways.

## 1 Introduction

Oral diseases remain among the most prevalent noncommunicable conditions worldwide. The Global Burden of Disease 2021 estimates that the main oral conditions affect approximately 3.69 billion people (1, 2), with the greatest burden concentrated among populations with limited access to affordable preventive and curative care (2). Untreated dental caries affects approximately 2.5 billion people in permanent dentitions and 514 million children with deciduous teeth (1, 2); severe periodontal disease affects more than one billion adults globally (1, 3); and lip and oral cavity cancers accounted for nearly 390,000 new cases and 188,000 deaths in 2022 (4). Survival also remains strongly stage-dependent, decreasing from 86.3% in localized oral cancer to 40.4% in distant disease in U.S. SEER data (5). These epidemiological patterns highlight a central problem in oral healthcare: the need for earlier detection, preventive follow-up, and timely referral is greatest in many of the same populations where access to dental professionals is most limited. Productivity losses attributable to caries reached US $22.46 billion for permanent teeth and US $1.55 billion for deciduous teeth in 2019 (6), while only 1.4% of the global dental workforce serves low-income countries (1). Expanding conventional in-person screening alone is therefore unlikely to close the prevention gap in many health systems without parallel digital, workforce, and public-health strategies.

Against this background, artificial intelligence (AI) has been proposed as a tool for extending oral-health screening and decision support beyond settings with regular specialist availability. The dental-AI literature has grown rapidly: a literature analysis of publications from 2011 to 2021 identified 1,497 articles on AI in dentistry, with annual growth averaging 21.6% over the decade and 34.9% over the final five years of that interval (7). Early applications were concentrated mainly in radiology, orthodontics, and general dental diagnostics (7). In caries detection, an umbrella review of seven systematic reviews reported that convolutional neural network (CNN)-based models achieved high sensitivity, specificity, and area-under-the-curve values across detection, segmentation, and classification tasks, with periapical and panoramic radiography yielding the strongest reported accuracy (8). A more recent overview of 116 systematic reviews on AI for dental diagnostics reported pooled sensitivity of 0.85 [95% confidence interval (CI), 0.76–0.93] and pooled specificity of 0.93 (95% CI, 0.90–0.95) (9). These findings indicate substantial technical progress in selected image-based diagnostic tasks, but they should not be interpreted as evidence that oral-health AI is already clinically mature across care settings.

The central challenge is that diagnostic performance under study conditions does not automatically translate into clinical benefit. The same literature that reports high accuracy also identifies important translational limitations, including risk of bias in primary studies, heterogeneous reference standards, limited external validation, and incomplete reporting of dataset composition. In periodontology, for example, there is still no agreed framework for clinical implementation, validation standards, or ethical governance of AI-based tools (10). Workforce preparedness is also incomplete: among medical, dental, and nursing students, pooled proportions for AI knowledge and attitudes were 0.44 and 0.65, respectively, across 22 studies and 8,491 participants (11). Teledentistry represents one of the digital infrastructures most likely to extend screening and triage, yet an overview of 30 systematic reviews found that only one directly addressed equity outcomes (12). The implementation gap is therefore not only algorithmic. It also involves validation standards, regulatory pathways, fairness auditing, clinician training, patient acceptability, affordability, and integration into care models that reach people who are not already receiving regular dental care.

In this review, precision prevention is defined as the use of multimodal, longitudinal, and context-sensitive data to identify dynamic oral-health risk trajectories and guide individualized preventive actions before disease progression occurs (13, 14). This concept differs from conventional preventive dentistry, which generally applies population-level or risk-category interventions such as oral-hygiene instruction, fluoride use, dietary counselling, and periodic recall (13–15). It also differs from precision medicine more broadly, which has often emphasized molecular stratification and individualized treatment selection after disease is established. In oral healthcare, precision prevention refers specifically to the integration of imaging, behavioral data, sensor-derived information, salivary or systemic biomarkers, clinical risk factors, and clinician oversight to support earlier detection, personalized prevention, timely referral, and longitudinal follow-up.

**Figure 1:**
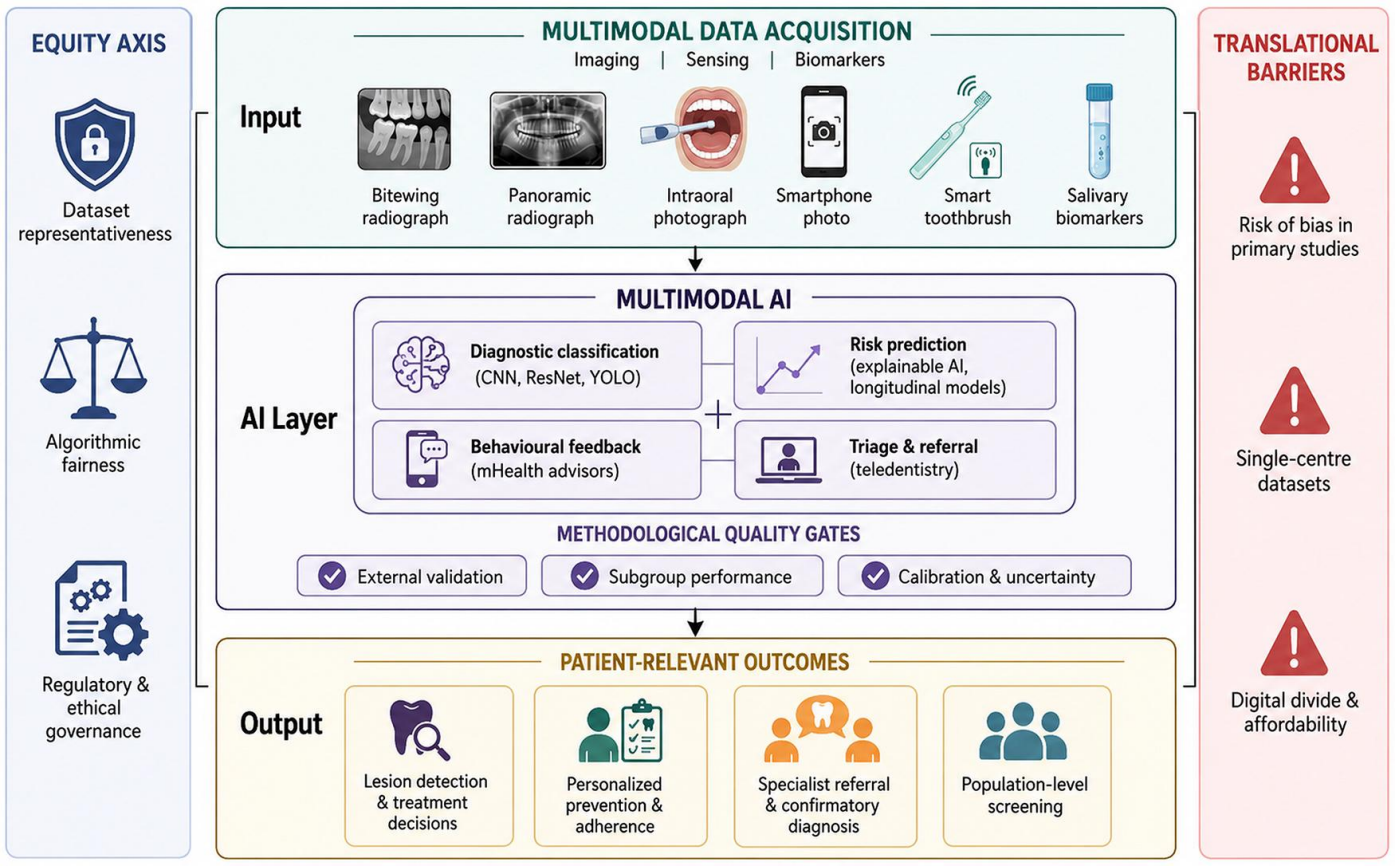
Conceptual framework for responsible multimodal artificial intelligence in oral healthcare. The figure summarizes the transition from remote screening to precision prevention through three linked operational tiers: multimodal data acquisition, AI-supported inference and decision support, and patient-relevant outcomes. Input data sources include radiographic imaging, intraoral and smartphone photography, sensor-based oral-hygiene monitoring, and salivary biomarkers. These inputs feed a multimodal AI layer that may support diagnostic classification, longitudinal risk prediction, behavioral feedback, and teledentistry-based triage or referral. Methodological quality gates, including external validation, subgroup performance assessment, calibration, and uncertainty estimation, are positioned before clinical output generation. Patient-relevant outputs include lesion detection and treatment decisions, personalized prevention and adherence support, specialist referral with confirmatory diagnosis, and population-level screening. The left-side equit*y* axis highlights dataset representativeness, algorithmic fairness, and regulatory or ethical governance as cross-cutting requirements, whereas the right-side panel summarizes recurrent translational barriers, including risk of bias, single-center datasets, and digital divide or affordability concerns (9, 12, 45, 48, 51, 56, 58). AI, artificial intelligence; CNN, convolutional neural network; mHealth, mobile health; OPMD, oral potentially malignant disorder; ResNet, residual neural network; YOLO, You Only Look Once.

Building on this operational definition, this narrative review examines the emerging role of AI in oral healthcare as the field moves from image-based remote screening toward precision prevention. It synthesizes evidence across caries detection, periodontal assessment and risk prediction, oral cancer and oral potentially malignant disorder (OPMD) screening, multimodal mobile-health interventions, teledentistry, and algorithmic fairness, using a conceptual framework that links multimodal data acquisition, AI-supported decision support, patient-relevant outcomes, and cross-cutting equity requirements (Figure 1). Particular attention is given to the distinction between clinical validity under study conditions and clinical value in real-world settings. The review emphasizes external validation, dataset representativeness, subgroup performance, downstream patient outcomes, regulatory preparedness, and equity-focused evaluation as central requirements for responsible implementation. Its aim is to clarify where oral-health AI appears clinically promising, where the evidence remains methodologically limited, and what conditions are needed for these technologies to support prevention without reinforcing existing disparities.

## 2 Methods

This narrative review was based on a structured literature search focused on artificial intelligence applications in oral-health screening, prevention, risk prediction, teledentistry, mobile health, and algorithmic fairness. A structured narrative design was selected because the objective was to synthesize conceptually and clinically heterogeneous evidence across diagnostic AI, risk prediction, multimodal mHealth, teledentistry, regulatory considerations, and equity, rather than to answer a narrowly defined intervention, exposure, or diagnostic-accuracy question suitable for quantitative pooling. This approach allowed integration of systematic reviews, diagnostic-accuracy studies, randomized trials, implementation studies, conceptual papers, and equity-focused literature into a prevention-oriented framework. Accordingly, the review emphasizes clinical interpretation, translational readiness, and implementation implications rather than exhaustive article-level capture or meta-analysis.

PubMed/MEDLINE was searched for English-language, peer-reviewed articles published from January 2022 through April 2026. Selected foundational articles published before 2022 were retained when they provided widely cited or conceptually important evidence directly relevant to the scope of the review.

Six thematic search domains were defined before evidence synthesis. Across all domains, search terms related to “artificial intelligence,” “machine learning,” and “deep learning” were combined with terms for: dentistry and oral health; dental caries, tooth decay, detection, diagnosis, prediction, and screening; periodontitis, periodontal disease, alveolar bone loss, diagnosis, screening, prediction, and risk; oral cancer, oral squamous cell carcinoma, mouth neoplasms, oral potentially malignant disorders, OPMD, and oral leukoplakia; mobile applications, smartphones, mobile health, mHealth, and multimodal oral-health technologies; and teledentistry, telehealth, telemedicine, equity, access, underserved populations, rural populations, low-income populations, bias, and health disparities. The search strategy combined Medical Subject Headings and free-text Title/Abstract terms, using Boolean operators adapted to each thematic domain. The complete PubMed search strategy by thematic domain is provided as Supplementary Table 1. The table reports the exact search strings, approximate domain-level search yields, and the number of records triaged within each domain to improve transparency; these counts were not deduplicated across domains and were not intended to constitute a PRISMA-style article-selection log.

Search filters were applied for publication date and human studies. Titles and abstracts were screened for relevance to AI applied to oral-health screening, prevention, diagnosis, risk prediction, mobile health, teledentistry, or equity. Full texts were prioritized when available, particularly for studies reporting diagnostic performance, clinical outcomes, implementation outcomes, or equity-related findings. Priority was given to systematic reviews, meta-analyses, randomized controlled trials, diagnostic-accuracy studies, cohort studies, and primary studies reporting clinically relevant performance or implementation outcomes. Narrative reviews were included when they addressed conceptual, ethical, regulatory, educational, or equity-related issues not adequately captured by quantitative studies.

Articles were excluded when they focused primarily on topics outside the scope of this review, including orthodontic appliance design, prosthodontic CAD/CAM workflows, non-oral telehealth applications, or administrative AI tools unrelated to oral-health screening or prevention. Editorials and commentaries without substantive primary or synthetic evidence were excluded. Articles were also excluded when sufficient methodological or outcome information could not be assessed from the abstract or accessible full text, or when they had been retracted.

This review was not conducted as a systematic or scoping review. No protocol was registered, no duplicate independent screening was performed, no formal risk-of-bias tool was applied across all included studies, and no quantitative meta-analysis was undertaken. Because a deduplicated article-level screening log with predefined exclusion reasons was not maintained, a PRISMA flow diagram was not generated. Instead, methodological transparency is provided through the structured description of the search domains, eligibility priorities, exclusion criteria, and Supplementary Table 1. The synthesis should therefore be interpreted as a structured narrative review designed to identify clinically relevant patterns, translational gaps, and implementation priorities, rather than as an exhaustive evidence map or pooled estimate of the available literature.

## 3 Landscape and clinical maturity

The dental-AI literature has expanded rapidly, but the evidence supporting routine clinical implementation has developed more slowly. A literature analysis of publications from 2011 to 2021 found that radiology accounted for the largest share of dental-AI activity, followed by orthodontics, general dental applications, restorative dentistry, and oral surgery (7). In caries detection, an umbrella review of seven systematic reviews reported that multilayer perceptrons, support vector machines, and convolutional neural networks had been applied across periapical radiographs, panoramic radiographs, smartphone images, bitewing radiographs, near-infrared transillumination, and other modalities, with periapical and panoramic imaging yielding the strongest reported accuracy (8). A broader overview of 116 systematic reviews reported pooled sensitivity of 0.85 and pooled specificity of 0.93 for AI-assisted dental diagnostic applications (9). These findings confirm substantial technical progress in selected image-based tasks, but they do not establish broad clinical readiness.

The distinction between diagnostic performance and clinical maturity is important. Many dental-AI studies still rely on single-center datasets, non-standardized outcomes, and performance metrics that are difficult to compare across tasks, imaging modalities, and populations (16). Dataset characteristics, annotation procedures, reference standards, and external validation methods are also inconsistently reported, limiting reproducibility and independent assessment. As a result, high accuracy in a development dataset should not be interpreted as evidence that a model will perform reliably across different devices, clinical workflows, demographic groups, or disease-prevalence settings. Although selected applications, particularly in orthodontics, appear closer to regulatory clearance or routine clinical use, most dental-AI tools remain at earlier translational stages (16, 17).

Clinical implementation also depends on the professionals who will use these systems. Available reviews emphasize that AI tools should support, rather than replace, dental professionals (17). This point is not merely rhetorical: clinicians must be able to interpret model outputs, recognize uncertain or out-of-distribution predictions, integrate AI-generated information with clinical findings, and explain uncertainty to patients. Current evidence suggests that educational preparedness may lag behind technical development. Among medical, dental, and nursing students, pooled proportions for AI knowledge and attitudes were 0.44 and 0.65, respectively, across 22 studies and 8,491 participants (11). Without training in model limitations, bias, calibration, and clinical interpretation, even technically strong tools may be implemented poorly or used in ways that increase rather than reduce clinical risk.

The field is also moving beyond isolated image classification. Reviews of AI in dental education and clinical practice describe potential roles in diagnostic information gathering, decision support, treatment planning, and patient-specific risk assessment (18, 19). In prevention-oriented applications, AI has been proposed for caries-risk prediction, personalized preventive recommendations, brushing-behavior monitoring, and adaptive feedback delivery (20). This shift is clinically important because the preventive value of oral-health AI is unlikely to come only from detecting lesions earlier; it may also depend on identifying risk trajectories, supporting behavior change, improving follow-up, and connecting patients to appropriate care pathways. These broader applications require evidence that extends beyond classifier accuracy to include patient adherence, treatment decisions, cost, safety, equity, and long-term oral-health outcomes.

Overall, the landscape literature describes a field with increasingly favorable diagnostic-performance estimates but uneven translational maturity. The more clinically relevant question is no longer whether AI systems can detect selected dental findings under study conditions, but whether their performance is reproducible across clinical settings, demographic groups, imaging devices, and models of care. This implementation question frames the disease- and pathway-specific evidence reviewed in the following sections: caries detection, periodontal disease assessment, oral cancer and OPMD screening, multimodal mHealth, teledentistry, and algorithmic fairness.

## 4 Caries detection

AI systems for caries detection have shown promising diagnostic performance across radiographic, near-infrared, and photographic modalities, but reported results vary substantially according to imaging method, lesion type, dataset composition, and validation design. A recent systematic review of deep learning applied to bitewing radiographs included 23 studies published before March 2025 and reported accuracies ranging from 70% to 99%, with ResNet and YOLO among the most frequently used architectures and dataset sizes ranging from 112 to 8,539 images (21). Another systematic review of 20 studies on AI-based caries lesion detection reported sensitivities from 0.44 to 0.86, specificities from 0.85 to 0.98, and AUC values from 0.84 to 0.98 (22). An umbrella synthesis of seven systematic reviews similarly found that convolutional neural network-based models generally achieved high sensitivity, specificity, and AUC across detection, segmentation, and classification tasks (8). In a randomized controlled trial of 200 intraoral radiographs, AI-based software reached sensitivity of 88%, specificity of 91%, and overall accuracy of 89%, compared with 84%, 88%, and 86%, respectively, for human interpretation (23). These findings indicate that deep-learning systems can approach clinician-level performance in selected caries-detection tasks, particularly under controlled radiographic study conditions.

The strength of this evidence is limited by important methodological concerns. In the recent bitewing systematic review, all 23 included studies were rated as having high risk of bias in at least one QUADAS-2 domain, especially in patient selection, index-test interpretation, and reference standards (21). The same review also reported substantial variation in model architectures, datasets, diagnostic metrics, and reporting standards (21). Broader dental-AI syntheses show similar uncertainty. An overview of 116 systematic reviews reported substantial heterogeneity for pooled sensitivity, specificity, AUC, and accuracy (9). These findings suggest that high accuracy in selected datasets should not be interpreted as evidence of consistent clinical reliability across populations, devices, diagnostic thresholds, or care settings.

Clinical utility also depends on how AI changes treatment decisions. The largest economic evaluation to date found that AI decision support for caries detection produced modest gains in tooth-retention years and slightly lower lifetime cost per patient in a German setting, but the results were highly sensitive to the fee charged for the AI service and to the treatment pathway followed after diagnosis (24). In an AI-aided versus unaided diagnostic trial on bitewing radiographs, AI assistance increased AUC from 0.85 to 0.89 and sensitivity from 0.72 to 0.81, while specificity remained unchanged (25). However, AI assistance was also associated with more invasive treatment decisions (25). This finding is clinically important because higher sensitivity may detect more early or ambiguous lesions, but it may also increase restorative intervention for enamel-stage lesions that might otherwise be managed preventively.

Performance also differs by imaging modality and clinical context. A systematic review of caries detection using oral photographs reported F1-scores of 68.3–95.4% for professional cameras, 78.8–87.6% for intraoral cameras, and 42.8%–80% for smartphone cameras (26). This suggests that consumer-grade imaging may underperform clinic-grade imaging, which is directly relevant to remote screening and teledentistry workflows. Pediatric and task-specific studies show similar variability. In pediatric dentistry, one meta-analysis reported accuracy ranging from 60% to 99%, sensitivity from 20% to 100%, and specificity from 49% to 100% across diagnostic and treatment-planning tasks (27). A separate review focused on early-childhood caries reported pooled sensitivity and specificity of 80% and 81%, respectively (28). This variability likely reflects differences in lesion definitions, dentition type, age group, imaging protocol, annotation quality, and reference standard.

More recent studies have broadened the scope of caries-related AI beyond binary detection. A deep-learning algorithm for staging secondary caries on bitewing radiographs reported specificity of 0.966 and AUC of 0.940, with a continuous severity score correlated at 0.802 with expert consensus (29). A systematic review and meta-analysis comparing AI applied to clinical photographs and bitewing radiographs reported pooled sensitivity of 76% and specificity of 91%, with clinical-image models showing higher sensitivity and similar specificity in caries detection (30). Clinical validation of a commercial convolutional neural network-based system on intraoral radiographs reported sensitivity, specificity, and diagnostic accuracy ranges of 0.51–0.76, 0.88–0.97, and 0.76–0.86 across observers, suggesting reproducible but more modest performance than that reported in some earlier development studies (31). A multicenter study with external testing across Germany, the Netherlands, and Slovakia reported F1-scores ranging from 0.749 for caries-lesion segmentation to 0.988 for root-canal-treatment identification, showing that external validation is feasible but performance remains highly task-dependent (32).

Overall, caries detection is one of the more technically advanced areas of dental AI, but its clinical value remains unresolved. The key question is no longer whether AI can detect caries in selected images, but whether AI-assisted workflows improve patient-relevant outcomes without increasing overtreatment. Future evaluations should therefore prioritize prospective clinical studies that measure treatment decisions, non-invasive management, restorative burden, tooth retention, cost-effectiveness, and patient experience. Until such evidence is available, AI for caries detection is best understood as a decision-support tool with promising diagnostic performance, not as a standalone screening or treatment-planning system.

## 5 Periodontal disease

Current periodontal-AI research can be grouped into two main areas: image-based detection of periodontal bone loss and longitudinal modeling of disease risk or treatment response. Image-based detection is relevant for screening and clinical assessment, whereas longitudinal prediction may be more directly aligned with precision prevention. This distinction is important because periodontal disease is not only a diagnostic state but also a chronic condition shaped by risk factors, behavior, systemic health, treatment adherence, and maintenance over time.

Radiographic AI models have shown promising performance for detecting periodontal bone loss and periodontitis. A recent systematic review using APPRAISE-AI quality scoring, together with a meta-analysis of ten studies on deep learning applied to panoramic and periapical radiographs, reported pooled sensitivity of 87% (95% CI, 80%–93%), specificity of 76% (95% CI, 69%–81%), and accuracy of 84% (95% CI, 75%–91%) (33). However, methodological quality remains uneven. Among the 30 included studies, 19 were rated as intermediate quality and only seven as high quality; no study reached very high quality on APPRAISE-AI (33). This suggests that radiographic periodontal AI has technical promise, but reporting quality, transparency, and validation standards remain insufficient for broad clinical implementation. A recent narrative review reached a similar conclusion, noting the absence of formal consensus or universally accepted guidelines for the clinical implementation, validation, and ethical governance of AI-based tools in periodontal diagnosis and treatment (10).

Non-radiographic approaches are also beginning to expand the periodontal-AI evidence base. A cross-sectional diagnostic-accuracy study used a multi-instance deep-learning model based on ResNet50 to classify stage II–IV periodontitis from a single frontal-view oral photograph. The study included 387 participants for internal model development and 183 participants for external testing in Shanghai (34). The model achieved an AUROC of 0.93 in both internal and external testing and showed higher sensitivity and specificity than clinicians of different skill levels in that study (34). Class-activation heat maps were highly consistent with periodontist annotations, suggesting that the model focused on clinically meaningful regions (34). Adding non-clinical parameters produced only marginal improvements, indicating that the frontal-view image carried most of the predictive signal in that specific setting (34). These findings are encouraging for remote screening, but broader validation is needed across different populations, cameras, lighting conditions, periodontal phenotypes, and clinical environments.

AI-supported digital periodontal assessment represents another emerging direction. PerioAI integrates intraoral scans with cone-beam computed tomography to support automated and non-invasive periodontal evaluation, with the potential to standardize assessment and reduce reliance on manual probing in selected contexts (35). This type of workflow may be useful when conventional periodontal charting is incomplete, inconsistent, or difficult to repeat at scale. However, its clinical role remains to be defined. Periodontal diagnosis still requires integration of clinical attachment levels, probing depths, bleeding, mobility, radiographic bone loss, risk factors, and longitudinal change. Digital assessment tools should therefore be evaluated as adjuncts to periodontal examination rather than replacements for clinical judgment.

Longitudinal prediction may be the most prevention-oriented application of periodontal AI. An explainable AI model trained on 1,012 variables from 30,465 NHANES participants achieved an AUC of 0.858 ± 0.011 for opportunistic screening and 0.865 ± 0.008 for periodontitis prediction in a diagnostic dataset (36). Age, sex, diabetes status, tissue transglutaminase, and smoking status emerged as the most important features, while arthritis, sleep disorders, hypertension, cholesterol, and overweight were identified as additional contributors (36). These findings are clinically plausible because periodontal risk is shaped by systemic, behavioral, inflammatory, and demographic factors. They also illustrate why prevention-oriented models may need to move beyond dental imaging alone.

Models predicting treatment response provide an additional step toward clinical decision support. A machine-learning model for one-year clinical response to periodontal treatment was trained on 414 South American patients from randomized controlled trials and externally tested on 78 patients from North America and Europe (37). Internal performance was high, with an AUROC of 0.93, and feature importance was distributed across clinical variables, treatment-related variables, microbiological variables, and demographic variables. External testing, however, decreased to an AUROC of 0.76 (37). This performance drop is important because it demonstrates the challenge of transporting periodontal models across populations, disease severities, treatment protocols, and microbiological profiles. A multicenter longitudinal study comparing logistic regression, multilayer perceptron, and a probabilistic graphical model for predicting periodontitis progression over 12 months found that the probabilistic graphical model performed best, combining clinical measures, salivary IL-1β, age, and sex to achieve an AUROC of 0.88, sensitivity of 0.55, and specificity of 0.81 (38). Together, these studies suggest that periodontal progression and treatment response are estimable from multimodal data, but external generalizability remains a central limitation.

The periodontal-AI evidence base therefore resembles the caries-AI literature in showing promising diagnostic performance alongside persistent concerns about study quality and validation. It differs, however, in its preventive potential. Periodontitis develops over years and can be monitored through repeated clinical measures, imaging, behavioral information, salivary biomarkers, microbiological profiles, and systemic health variables. This makes it a strong candidate for AI models that estimate risk trajectories, personalize maintenance intervals, and identify patients who may benefit from intensified prevention or closer follow-up. The key question is not only whether AI can detect periodontal disease, but whether AI-guided care can improve attachment stability, reduce inflammatory burden, preserve teeth, improve adherence, and support long-term maintenance.

Future periodontal-AI studies should prioritize external validation, representative cohorts, standardized periodontal case definitions, longitudinal outcomes, and integration into preventive care pathways. Models should be tested across different populations, disease severities, imaging devices, and clinical settings. They should also report subgroup performance, calibration, clinical decision impact, and patient-relevant outcomes. Until such evidence is available, periodontal AI should be considered a promising decision-support and risk-stratification tool, not a standalone diagnostic or preventive system.

## 6 Oral cancer and oral potentially malignant disorders

AI-assisted screening for OPMD and oral cancer has reported some of the highest diagnostic-performance estimates in oral-health AI, particularly when applied to clinical photography. A recent meta-analysis of AI-assisted clinical imaging for OPMD and oral cancer screening reported an overall diagnostic odds ratio of 68.4, an area under the summary receiver operating characteristic curve of 0.938, sensitivity of 89.9%, and specificity of 89.2% across the included studies (39). After Graphic Display of Heterogeneity sensitivity analysis, the adjusted diagnostic odds ratio was 49.3, with an area under the summary receiver operating characteristic curve of 0.877, sensitivity of 90.5%, and specificity of 87.0% (39). Clinical photography showed the highest diagnostic accuracy among the imaging modalities assessed (39). This is clinically relevant because photographic screening could support recognition and referral in settings where specialist oral-medicine expertise is limited, provided that image quality, referral capacity, and confirmatory diagnostic pathways are available.

These performance estimates are promising, but they should not be interpreted as direct evidence of population-level screening effectiveness. Much of the available evidence comes from selected or referral-based datasets rather than from general screening populations. This distinction is important because OPMD and oral cancer prevalence is usually lower in community screening settings than in enriched clinical datasets. In low-prevalence populations, even models with high sensitivity and specificity may generate a substantial number of false-positive referrals, increasing the burden on confirmatory diagnostic services. Therefore, the central translational issue is not only whether AI can classify suspicious lesions accurately, but whether it can improve timely referral and diagnosis without overwhelming clinical pathways or worsening inequities in access.

A complementary systematic review of deep-learning models for oral cancer detection reported accuracies ranging from 85% to 100%, with a pooled diagnostic odds ratio of 2,549 for classification subgroups (40). These estimates are striking, but they should be interpreted cautiously because very high diagnostic odds ratios may reflect selected datasets, limited external validation, or narrow classification tasks rather than robust performance in real-world screening. Reviews of AI applied to oral epithelial dysplasia and oral squamous cell carcinoma have also expanded the evidence base beyond clinical photography to include pathomics, epigenomics, multiplex immunohistochemistry, and deep-learning digital pathology (41). These approaches may support histopathological classification, risk stratification, and molecular characterization, but they are closer to specialist diagnostic support than to community-level remote screening.

Domain shift is another major limitation. A Bayesian deep-learning model using Probabilistic HRNet with uncertainty estimation achieved an F1-score of 64.9% and AUC of 80.3% on a domain-shift test set; performance improved to an F1-score of 74.4% and AUC of 85.6% after redirecting the 30% most uncertain samples to expert review (42). This finding is important because it illustrates two points. First, model performance may decline when images differ from the development dataset in acquisition conditions, population characteristics, lesion appearance, or clinical setting. Second, uncertainty-aware workflows may be useful for triage because they allow uncertain cases to be routed toward expert assessment rather than classified automatically. In oral cancer screening, this type of human-in-the-loop design may be safer and more clinically realistic than fully automated diagnosis.

Clinical-photography models should also be compared with the screening pathways in which they are expected to operate. In a multicenter study using 5,576 endoscopic images from five Korean university hospitals, a deep-learning model for tongue cancer diagnosis achieved sensitivity, specificity, and accuracy of 81.1%, 86.8%, and 84.7%, respectively (43). The model outperformed general physicians, who achieved 77.3%, 75.0%, and 75.9%, but remained below oncology specialists, who achieved 91.7%, 90.9%, and 91.2% (43). This comparison is clinically meaningful because AI-supported screening is most likely to be useful in settings where specialists are unavailable. The goal, therefore, should not be to replace specialist evaluation, but to support generalist assessment, identify lesions requiring referral, and reduce diagnostic delay.

AI has also been investigated for prognostic and predictive tasks in oral cancer. An MRI-based ResNet50 model combining radiomics and deep-learning features predicted occult cervical lymph-node metastasis in early oral and oropharyngeal squamous cell carcinoma with an AUC of 0.928 in the training set, decreasing to 0.878 in the test set and 0.796–0.834 in two external validation sets (44). If prospectively validated, such models could help inform risk stratification for elective neck dissection, but the current evidence is not sufficient to guide treatment decisions independently. A deep radiomics framework combining optical coherence tomography images of oral mucosa with peripheral-blood immune indicators in 68 patients, including 1,445 OCT images, predicted oral cancer prognosis with an AUC of 0.886 and identified the systemic immune-inflammation index as the most informative single feature (45). At the histological level, a graph convolutional network trained on cell spatial organization in tissue images identified reduced structural concordance and increased epithelial-substratum closeness as features associated with elevated risk of malignant transformation in OPMD (46). These studies suggest that AI may contribute not only to lesion detection but also to prognostic assessment, malignant-transformation risk, and individualized oncologic decision support.

Despite these advances, AI-supported detection alone is unlikely to improve oral-cancer outcomes unless it is embedded within a complete screening, referral, and treatment pathway. A broader review of novel diagnostic techniques for oral cancer has placed AI alongside liquid biopsy, next-generation sequencing, microarray, nanotechnology, and lab-on-a-chip platforms (47). This wider context is important because survival gains depend on more than diagnostic technology. Earlier presentation, accurate referral, histological confirmation, timely specialist treatment, and access to follow-up care remain essential. AI may help identify suspicious lesions, but it cannot compensate for absent referral infrastructure, unaffordable diagnostic confirmation, or delayed treatment access.

Overall, AI-assisted oral cancer and OPMD screening is clinically promising but not yet established as a population-level screening solution. Clinical photography combined with AI may support generalist assessment and referral under selected and validated conditions (39). Prognostic and multimodal models may also contribute to risk stratification in specialist settings (44–46). However, future studies must move beyond selected datasets and diagnostic metrics. Priority should be given to prospective validation in target screening populations, reporting of disease prevalence and positive predictive value, subgroup and domain-shift evaluation, uncertainty-aware referral workflows, and linkage from AI-positive findings to definitive diagnosis and timely treatment. Until such evidence is available, AI should be viewed as a triage and decision-support tool for oral cancer screening rather than as an autonomous diagnostic system.

## 7 Multimodal AI and mHealth

Some of the most clinically relevant evidence for preventive AI applications in dentistry comes from interventions that combine sensing, image capture, behavioral feedback, and clinician support rather than from classifier performance alone. This distinction is important because prevention depends not only on detecting disease, but also on changing behavior, improving adherence, monitoring risk over time, and linking patients to appropriate follow-up. In this context, mHealth platforms and AI-enabled devices may offer greater preventive value when they are embedded into daily routines rather than limited to episodic clinic-based diagnosis.

Randomized evidence suggests that AI-enabled feedback systems can improve selected periodontal outcomes when integrated with patient monitoring and clinician-directed support. In one trial, 100 patients with generalized stage II/III periodontitis were randomized to either a standard oral-hygiene regimen or an AI-enabled multimodal-sensing toothbrush coupled with targeted mHealth micromessages from the clinical team (48). At six months, the proportion of inflamed periodontal pockets decreased from 80.7% to 52.3% in the control group and from 81.4% to 44.4% in the intervention group, yielding an inter-group difference of 7.9 percentage points. The intervention group also achieved better oral-hygiene levels, with no significant adverse events reported (48). These findings suggest that AI may add clinical value when used to support repeated behavioral feedback and clinician follow-up rather than as a stand-alone diagnostic classifier.

A second randomized trial provides related evidence using consumer-facing hardware. In that study, 150 participants were assigned to an interactive telemonitoring toothbrush, an oscillating-rotating power toothbrush, or a manual toothbrush and followed for six months (49). The Simple Hygiene Score increased in the manual-toothbrush group but decreased in the interactive-telemonitoring group, while the Quigley-Hein plaque index also decreased in the telemonitoring arm (49). In addition, 92% of users in the AI-enabled arm reported improved brushing ability, compared with 76% in the power-toothbrush arm (49). Although differences in populations, comparators, devices, and endpoints limit direct comparison between trials, both studies support the possibility that AI-enabled monitoring may improve oral-hygiene behavior when feedback is delivered repeatedly and linked to user action.

Smartphone-based oral-health applications and AI advisors extend this prevention model beyond smart toothbrushes. A scoping review of 45 studies on patient-oriented mobile-phone applications in oral health, including 23 randomized controlled trials, found that nine trials reported substantial reductions in plaque and six reported significant improvements in gingival health (50). A focused systematic review of AI-powered oral-health self-monitoring using smartphone selfies retained four studies after screening 1,639 records and 64 full texts (51). Short-term use, up to six months, was associated with statistically significant reductions in plaque and gingival index scores, and combining the AI advisor with verbal counseling appeared to enhance effectiveness (51). Across these interventions, the recurring model is similar: a smartphone, smart toothbrush, or related device captures behavioral or image data, an AI system generates personalized feedback, and the patient receives guidance intended to improve oral-hygiene behavior. The magnitude and durability of benefit remain uncertain, but the available evidence suggests that repeated feedback may be more clinically relevant than one-time automated assessment.

Clinical deployment studies also illustrate both the promise and limitations of AI-enabled mHealth. A prospective study of 191 consecutive endodontic patients evaluated 4,361 teeth using an intraoral camera combined with an AI model based on MobileNet-v3 and U-Net architectures (52). Overall accuracy reached 93.40%, with sensitivity of 81.31%, specificity of 95.65%, and positive predictive value of 77.68% (52). However, performance varied substantially by tooth position and lesion type. Accuracy was highest in anterior teeth, while sensitivity was close to 10% for interproximal lesions in anterior teeth and buccal lesions in premolars (52). This finding is important for remote screening because a high overall accuracy can conceal clinically meaningful failure modes in specific anatomical sites or lesion categories.

The application space for AI-enabled mHealth also extends beyond caries detection. A review of mobile applications in oral cancer screening identified nine studies among 423 records and reported that mobile applications with specific algorithms have been used for risk assessment, screening, diagnosis of oral potentially malignant and malignant disorders, remote consultation, referral, treatment support, self-monitoring, and medication adherence (53). This broader use case is clinically relevant because mHealth platforms may support multiple stages of preventive and diagnostic care: identifying risk, prompting screening, facilitating remote review, supporting referral, monitoring adherence, and maintaining follow-up. However, these functions require validation within care pathways, not only demonstration that an app or algorithm can classify images.

Taken together, the multimodal AI and mHealth literature suggests that AI offers preventive value mainly when embedded in patients’ daily routines and connected to clinical follow-up. Trials of AI-enabled toothbrushes and smartphone feedback systems indicate reductions in inflamed periodontal pockets, plaque indices, and gingival inflammation over six-month follow-up (48, 49, 51). The evidence base, however, remains small, and durability, scalability, cost-effectiveness, and equity are not yet established. Populations with the highest preventive need may also face the steepest barriers to mHealth implementation, including device cost, internet access, digital literacy, data-privacy concerns, and limited integration with dental or primary-care services.

A plausible model of multimodal precision prevention is therefore beginning to emerge. AI could integrate images, behavioral sensor data, salivary biomarkers, and longitudinal risk models to identify patients at elevated risk and deliver personalized preventive support. Related work in salivary biomarkers and multi-omics suggests that AI may help operationalize biomarker panels across periodontal disease, caries, oral cancer, temporomandibular disorders, and salivary gland diseases (54). Whether this model can be delivered equitably will depend on the infrastructure built around it: affordability, accessibility, data governance, clinician oversight, interoperability, and reliable linkage to preventive care. Until these conditions are met, AI-enabled mHealth should be viewed as a promising prevention-support strategy, not as a self-contained solution for oral-health inequities.

## 8 Teledentistry, equity, and algorithmic fairness

Teledentistry has expanded rapidly, but evidence on whether it reduces or widens access disparities remains limited. This gap becomes more consequential as AI tools are increasingly proposed for remote screening, triage, diagnosis, monitoring, and patient communication. An umbrella review of systematic reviews and meta-analyses identified 1,020 records and retained 30 systematic reviews, with individual reviews including between 130 and 7,913 participants (12). Effectiveness was addressed in 22 of 30 reviews, patient-centered care in 14, and efficiency in 11. Equity, by contrast, received direct attention in only one review, while broader concerns about reducing inequities in vulnerable populations appeared in nine reviews (12). Patient safety appeared in eight reviews, data privacy in three, and confidentiality in two. Methodological quality was also limited: 25 of 30 reviews were rated as critically low to low quality, and eight had high risk of bias (12). This pattern suggests that teledentistry research has prioritized effectiveness more than equity, safety, privacy, or confidentiality, despite the importance of these domains for remote and AI-enabled care.

The central implementation issue is conditional access. Teledentistry may expand care only when the underlying digital, financial, and clinical infrastructure is available. AI may support remote screening, diagnosis, record-keeping, and patient monitoring, and may contribute to a shift from reactive treatment toward more preventive and personalized oral healthcare (55). However, the barriers are often concentrated in the populations most likely to benefit from remote care, including communities with limited internet coverage, high equipment costs, low digital literacy, and reduced access to follow-up services (56). When these barriers are reduced, acceptability can be favorable. In the ORIGINS Project in Western Australia, a cohort study of 42 primary caregivers using a teledental screening application reported high perceived ease of use, perceived usefulness, and behavioral intention (57). These findings suggest that teledentistry can be acceptable under supportive conditions, but they do not establish generalizability across underserved populations or health systems with different infrastructure.

Older adults illustrate this implementation tension clearly. Geriatric oral health often requires longitudinal care, caregiver involvement, and adaptation to functional, cognitive, financial, and technological barriers. Work in geriatric oral health has emphasized the need for co-designed studies grounded in real-world service delivery, noting that conventional randomized-trial frameworks may be difficult to apply in vulnerable older populations and that longitudinal acceptability and outcomes data for telehealth remain limited (58). Pilot success in digitally supported populations therefore should not be assumed to translate automatically across age groups, socioeconomic contexts, rural settings, or communities with limited access to dental services.

Algorithmic fairness introduces an additional concern for AI-enabled teledentistry. Many oral and craniofacial AI models are trained on relatively homogeneous clinical datasets in which historically disadvantaged demographic groups, including ethno-racial minorities, are under-represented (59). When models trained on such datasets are deployed in populations that differ from the development population, subgroup performance may be lower and existing inequities may be reproduced or amplified (59). This is a practical clinical concern rather than only an ethical abstraction. Model performance depends on the relationship between training data, deployment population, imaging conditions, device type, disease prevalence, and clinical workflow. Without transparent reporting of dataset composition and subgroup performance, it is difficult to determine whether an AI tool performs equitably across the populations in which it will be used.

Recent dataset development illustrates both progress and remaining limitations. A publicly available annotated intraoral image dataset for AI-driven dental caries detection included 6,313 images collected from individuals aged 10 to 24 years in Mithi, Sindh, Pakistan, and YOLOv8s achieved a mean average precision of 0.841 at an intersection-over-union threshold of 0.5 (60). The inclusion of data from a low- and middle-income setting is important, particularly because many dental-AI datasets have historically relied on high-income clinical populations. However, image acquisition with a single mobile device also limits generalizability. Even when demographic representation improves, restricted device diversity, imaging protocols, lighting conditions, and metadata can still constrain external performance.

A second strand of fairness research situates AI in oral health within the United Nations Sustainable Development Goals framework. AI may reduce transportation needs, optimize care delivery, expand service accessibility, and contribute to reduced inequality in some contexts. At the same time, biased, inaccessible, unaffordable, or poorly governed systems may reinforce inequality and discrimination (61). Privacy, confidentiality, security, resource requirements, and implementation costs are particularly relevant for digital and AI-enabled oral-health systems (61). These concerns are amplified in remote screening because patients may be asked to share images, behavioral data, biomarker information, or clinical histories through consumer devices and digital platforms.

Overall, teledentistry and AI-enabled remote care should be evaluated as pathway-level interventions rather than as isolated technologies. Their value depends not only on diagnostic accuracy or user acceptability, but also on affordability, digital access, data governance, clinician oversight, referral completion, confirmatory diagnosis, and continuity of care. For remote screening tools, future studies should report performance across demographic groups, imaging devices, acquisition settings, and deployment environments whenever feasible. They should also evaluate what happens after an AI-positive result, including whether patients complete referral, receive timely specialist assessment, obtain confirmatory diagnosis, and enter appropriate treatment or prevention pathways. Until these conditions are addressed, AI-enabled teledentistry should be considered a promising but equity-sensitive strategy, not an automatic solution to oral-health disparities.

## 9 Discussion

The evidence reviewed across clinical and preventive domains indicates that oral-health AI has achieved encouraging diagnostic performance in selected image-based tasks, particularly caries detection, periodontal bone-loss assessment, and OPMD/oral-cancer screening. However, diagnostic accuracy under study conditions should not be equated with clinical readiness. Across the reviewed domains, translation remains constrained by risk of bias in primary studies, heterogeneous reference standards, limited external validation, incomplete demographic reporting, uncertain downstream clinical effects, and insufficient integration of equity, regulation, and care pathways. The relevant question is therefore not only whether AI can classify oral-health findings accurately, but whether AI-supported workflows improve patient-relevant outcomes in diverse real-world settings. Table 1 summarizes the strongest direction-of-effect signal reported in each clinical domain alongside its methodological quality profile and the key translational barriers identified across the reviewed literature. The pattern across domains is consistent: performance estimates are favourable under study conditions, yet methodological quality, dataset representativeness, and downstream pathway integration remain limiting factors in every domain.

**Table 1:** Cross-domain summary of reported AI performance, methodological quality, and translational barriers in prevention-oriented oral healthcare.

Clinical domain	Best-reported diagnostic performance	Methodological quality	Key translational barriers
Caries detection	Pooled sensitivity 0.85; specificity 0.93 (overview of 116 SRs) (9); AI-aided RCT: AUC 0.89 with AI assistance vs 0.85 unaided; sensitivity increased 0.72 to 0.81 (25)	All 23 included studies in bitewing SR rated high RoB in ≥1 QUADAS-2 domain (21); pooled I^2^ = 75–98% across metrics (9)	Increased invasive treatment decisions despite higher sensitivity (25); cost-effectiveness highly sensitive to AI fee and downstream pathway (24); smartphone-grade imaging F1 42.8%–80% vs 68.3–95.4% professional cameras (26)
Periodontal disease	Pooled sensitivity 0.87 (95% CI 0.80–0.93); specificity 0.76 (95% CI 0.69–0.81); accuracy 0.84 (95% CI 0.75–0.91) (33); photographic AUROC 0.93 internal and external (34)	Only 7/30 studies (23.3%) reached high quality on APPRAISE-AI; no study very high quality (33)	External AUROC drops from 0.93 to 0.76 across populations (37); absence of consensus on validation, governance, ethical implementation (10); longitudinal cohorts limited
Oral cancer & OPMD	Diagnostic odds ratio 68.4 (95% CI 39.5–118.6); SROC AUC 0.938; sensitivity 89.9%, specificity 89.2% (39); tongue cancer model 81.1/86.8/84.7% sens/spec/acc (43)	High heterogeneity and inconsistent reference standards, including histopathology, expert visual assessment, and consensus diagnosis (39, 41); performance generated mostly from referral-based samples	Domain-shift with F1-score improving from 64.9% to 74.4% only after expert review of uncertain cases (42); AI outperformed generalists but remained below specialists (43); biomarker absence for individualized risk stratification (41)
Multimodal AI & mHealth	RCT inflamed-pocket reduction 7.9 percentage points at 6 months (95% CI 1.6–14.6; *P* < 0.05) (48); intraoral-camera AI accuracy 93.40%, but sensitivity ∼10% interproximal anterior lesions (52)	Single-centre RCTs (*n* = 100, *n* = 150); evidence base small; risk-of-bias moderate-to-low across 4 selfie-AI studies (51)	Performance highly variable by anatomical site (52); durability, scalability, cost-effectiveness unestablished; populations with greatest preventive need face highest mHealth barriers (device cost, connectivity, literacy)
Teledentistry, equity & fairness	YOLOv8s mAP 0.841 on Sindh (Pakistan) intraoral dataset (60); user acceptance 4.54–4.65 (out of 5) in caregiver cohort (57)	Most teledentistry reviews had low methodological confidence: 25/30 were rated critically low to low, and 8/30 had high risk of bias (12)	Equity directly addressed in only 1/30 reviews (3%) (12); ethno-racial minorities remain under-represented in training datasets (59); single-device acquisition may restrict dataset generalizability (60)

AI, artificial intelligence; AUC, area under the curve; AUROC, area under the receiver operating characteristic curve; CI, confidence interval; mAP, mean average precision; OPMD, oral potentially malignant disorders; RCT, randomized controlled trial; RoB, risk of bias; SR, systematic review; SROC, summary receiver operating characteristic.

The domains emphasized in this review were selected because they map directly onto the proposed transition from remote screening to precision prevention. Caries detection, periodontal assessment, oral cancer and OPMD screening, multimodal mHealth, teledentistry, and algorithmic fairness each involve clinically meaningful links between early detection, risk stratification, patient follow-up, referral pathways, and equitable access. Other areas of dental AI, including orthodontic diagnosis and treatment planning, image segmentation, prosthodontic CAD/CAM workflows, and appliance design, were not reviewed in detail because they were outside this prevention- and screening-focused scope, not because they are less mature or less relevant to dental AI. This domain selection was therefore intended to prioritize applications in which AI may alter prevention-oriented care pathways and population-level access.

Across domains, the central pattern is consistent: reported diagnostic performance is often favorable, but clinical consequences remain insufficiently characterized. In caries detection, AI assistance may increase sensitivity, yet it may also shift treatment thresholds toward more invasive decisions for early or ambiguous lesions (21, 23, 25). In periodontology, radiographic and photographic models show promising performance, while longitudinal models suggest potential for risk prediction and treatment-response estimation (25, 33–38). However, methodological quality, implementation consensus, and transportability across populations remain limited (10, 33, 37). In oral cancer and OPMD screening, clinical-photography models report high pooled diagnostic accuracy (39), but population-level screening value remains uncertain because prevalence, positive predictive value, domain shift, referral completion, and access to confirmatory diagnosis are not fully resolved (39, 42, 43). These examples reinforce that AI performance must be interpreted in relation to downstream clinical decisions, not as an isolated technical endpoint.

This distinction between model performance and clinical utility is especially important for AI-enabled screening and prevention. Model performance describes how well an algorithm detects or predicts a target under defined study conditions; clinical utility requires evidence that its use improves decisions, safety, efficiency, access, or patient outcomes within a real care pathway. For oral-health AI, the consequences of an AI-positive result depend on whether patients receive appropriate advice, complete referral, obtain confirmatory diagnosis, initiate treatment when indicated, and remain connected to follow-up care. Without this pathway-level evidence, high diagnostic accuracy may overstate the practical value of an AI system.

Multimodal and mHealth applications may offer a more prevention-oriented model than isolated image classifiers. Trials of AI-enabled toothbrushes and smartphone-based feedback systems suggest that repeated monitoring, behavioral feedback, and clinician support can reduce periodontal inflammation, plaque indices, and gingival inflammation over short-term follow-up (48, 49, 51). This evidence is still limited, but it points toward a clinically meaningful direction: AI may be most useful when it supports repeated preventive behaviors and care-team interaction rather than when it delivers a one-time classification. The integration of imaging, behavioral sensors, salivary biomarkers, and longitudinal risk models could support precision prevention, but this promise will depend on durability of effect, affordability, data governance, clinician oversight, and linkage to preventive care (48, 51, 54).

Teledentistry presents a related implementation challenge. AI-supported remote screening may improve access for geographically isolated or underserved populations (55), but it may also exclude patients with limited internet access, low digital literacy, financial constraints, privacy concerns, or poor access to follow-up care (56). An overview of 30 systematic reviews found that equity was directly addressed in only one review, despite its central relevance to remote oral healthcare (12). At present, evidence is insufficient to determine whether AI-integrated teledentistry primarily democratizes dental care or further stratifies access. Its impact will depend on cost, device availability, data security, digital literacy, subgroup performance, and the strength of referral pathways for in-person diagnosis and treatment.

### 9.1 Fundamental methodological issues

Several methodological issues recur across the evidence base. Risk of bias and inconsistent reporting remain common. In caries detection, a systematic review of deep learning on bitewing radiographs rated all 23 included studies as having high risk of bias in at least one QUADAS-2 domain (21). In periodontology, only seven of 30 studies in a systematic review of AI for periodontal bone loss reached high quality on APPRAISE-AI, and none achieved very high quality (33). In teledentistry, 25 of 30 systematic reviews were rated as critically low to low methodological quality (12). These findings indicate that reported technical performance often exceeds the strength of the underlying methodological evidence.

External validation and calibration remain insufficient. An overview of 116 systematic reviews on AI in dentistry reported substantial heterogeneity in pooled sensitivity, specificity, AUC, and accuracy estimates (9), limiting confidence in generalizability. A multicenter external-testing study in caries detection found F1-scores ranging from 0.749 for caries-lesion segmentation to 0.988 for root-canal-treatment identification when models trained on bitewings from Germany and the Netherlands were tested externally on bitewings from Slovakia (32). This variation illustrates that even controlled external validation can reveal important task-specific performance differences. Similar concerns arise in oral cancer AI, where domain-shift testing showed performance reductions that improved only when uncertain cases were redirected to expert review (42). These findings support the need for external validation, calibration analysis, uncertainty estimation, and post-deployment monitoring before broad implementation.

Reference-standard heterogeneity also complicates interpretation, particularly in OPMD and oral cancer detection. Studies may use histopathological confirmation, specialist visual diagnosis, expert consensus, or combinations of these approaches. Such variability may influence pooled estimates and limit comparisons across studies (39). Reviews of AI for oral epithelial dysplasia and oral squamous cell carcinoma have also noted the absence of consistently reliable clinical, histological, molecular, or prognostic biomarkers for individualized risk stratification (38). Until reference standards and reporting practices become more consistent, diagnostic-performance estimates in this area should be interpreted cautiously.

### 9.2 Research gaps

Several gaps should guide future research. Prospective screening and implementation studies are needed. Diagnostic-accuracy evidence is now sufficient to support carefully designed prospective evaluation in domains such as caries detection, periodontal bone-loss assessment, and OPMD/oral-cancer screening (21, 22, 33, 39). However, relatively few studies have examined whether AI-supported workflows improve outcomes in the populations where these conditions are most prevalent. For screening applications, future studies should assess not only lesion detection, but also referral completion, diagnostic confirmation, treatment initiation, patient adherence, cost, and long-term clinical outcomes.

Dataset representativeness also remains insufficient. Fairness work in oral and craniofacial AI consistently shows that historically disadvantaged groups, including ethno-racial minorities, are under-represented in development datasets (59). Recent contributions from low- and middle-income settings, such as annotated intraoral image datasets from underserved regions, represent genuine progress, although single-device acquisition and limited metadata may still constrain generalizability (60). Future dataset reports should describe demographic composition, socioeconomic context when available, imaging hardware, acquisition setting, annotation procedures, and subgroup performance.

Longitudinal outcome and cost data remain sparse. The available cost-effectiveness analysis for AI-supported caries detection depends on assumptions about downstream treatment decisions and is highly sensitive to the fee paid for AI services (24). Real-world comparisons of AI-aided and standard pathways are needed to evaluate tooth retention, restorative burden, periodontal stability, referral completion, biopsy confirmation, treatment timeliness, patient adherence, and cost-effectiveness. Without such data, deployment decisions may rely too heavily on diagnostic metrics that are not directly linked to patient benefit.

Regulatory and equity frameworks also remain underdeveloped. Periodontology lacks formal consensus on implementation, validation standards, and ethical governance of AI-based tools (10). Teledentistry reviews seldom address equity directly, despite the centrality of access, privacy, safety, and follow-up outcomes for remote care (12). Responsible deployment will require evidence that AI systems perform not only accurately, but also safely, fairly, and usefully within real care pathways.

### 9.3 Future directions

Future oral-health AI research should move from isolated diagnostic performance toward integrated models of prevention, validation, and implementation. Multimodal, closed-loop preventive interventions are likely to be particularly important. In one randomized trial, an AI-enabled multimodal-sensing toothbrush combined with targeted mHealth micromessages produced a 7.9 percentage-point inter-group difference in inflamed-pocket reduction at six months (48). This suggests that AI may add clinical value when used to support behavior change and clinician follow-up rather than as a stand-alone classifier. Similar models should be tested in larger, more diverse, and longer-term trials across caries prevention, periodontal maintenance, oral-hygiene adherence, and OPMD self-monitoring.

Fairness should be incorporated from model development through implementation. Future studies should report performance stratified by demographic group, imaging device, dataset source, and deployment setting whenever feasible. Dataset publications should describe acquisition conditions and annotation processes in sufficient detail to permit external assessment. Fairness auditing is especially important for tools intended for remote screening or underserved populations (59).

Co-design with intended users is also essential. Pilot studies may report high perceived usefulness and ease of use when technologies are accessible (57), but underserved populations remain underrepresented in development and evaluation. Co-design with rural communities, older adults, low-income groups, ethno-racial minorities, caregivers, and frontline dental or primary-care providers may help align AI-enabled tools with real-world constraints, including digital access, health literacy, cultural acceptability, referral pathways, and privacy concerns (58–60).

Clearer regulatory and evaluation pathways are needed to connect technical performance with clinical benefit. Future frameworks should require external validation, demographic transparency, subgroup-performance reporting, workflow integration, data governance, and patient-relevant outcomes. For screening tools, evaluation should include what happens after an AI-positive result, including referral completion, specialist review, confirmatory diagnosis, treatment initiation, and follow-up. Without such pathway-level evaluation, the gap between diagnostic accuracy and clinical benefit will remain difficult to close.

### 9.4 Implementation, governance, and equity safeguards

**Figure 2:**
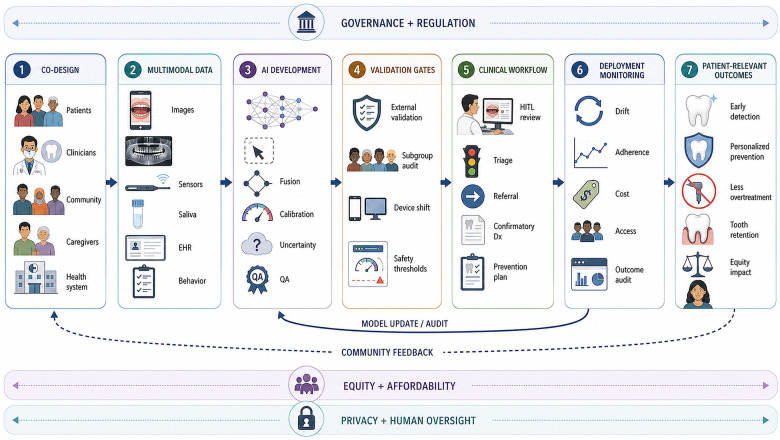
Responsible implementation pathway for multimodal AI-enabled precision prevention in oral healthcare. The figure illustrates a lifecycle approach in which AI-enabled oral-health tools move from co-design and multimodal data acquisition to model development, external validation, fairness auditing, clinical workflow integration, deployment monitoring, and patient-relevant outcomes. Multimodal inputs may include intraoral or smartphone photography, radiographs, sensor-derived oral-hygiene data, salivary or systemic biomarkers, electronic health records, behavioral information, and clinical risk factors. Before deployment, models should pass validation gates that assess calibration, uncertainty, device shift, subgroup performance, and safety thresholds. In clinical use, human-in-the-loop workflows should connect AI-supported triage to clinician review, referral, confirmatory diagnosis, and individualized prevention plans. Post-deployment monitoring should evaluate model drift, adherence, affordability, access, false-positive and false-negative patterns, and equity impact (62–68). AI, artificial intelligence; Dx, diagnosis; EHR, electronic health record; HITL, human-in-the-loop; mHealth, mobile health; OPMD, oral potentially malignant disorder; QA, quality assurance; SaMD, software as a medical device.

Responsible implementation of oral-health AI will require governance standards that extend beyond diagnostic accuracy. Depending on jurisdiction and intended use, AI tools used for screening, diagnosis, triage, or treatment support may fall within medical-device or software-as-a-medical-device regulatory pathways. Regulatory expectations are increasingly converging around risk management, transparent user information, human oversight, high-quality datasets, validation evidence, and post-deployment monitoring (62, 63). For oral-health AI, these requirements are particularly important because many proposed applications involve remote image acquisition, patient-facing mHealth platforms, automated triage, and referral decisions in populations with unequal access to dental care. Figure 2 summarizes a practical implementation pathway for multimodal AI-enabled precision prevention, emphasizing that responsible deployment requires co-design, representative data, external validation, fairness auditing, human oversight, clinical referral pathways, and post-deployment monitoring.

Several practical safeguards should be incorporated before deployment. First, models should undergo external validation in the intended population and care setting, with reporting of calibration, uncertainty, and subgroup performance by demographic characteristics, imaging device, acquisition setting, and disease prevalence whenever feasible. Second, fairness audits should evaluate whether error rates, false-positive referrals, false-negative findings, and model confidence differ across clinically or socially relevant groups. Third, human-in-the-loop workflows should be used for high-risk outputs, especially oral cancer and OPMD screening, so that uncertain or positive findings trigger clinician review, confirmatory diagnosis, and referral rather than autonomous decision-making. Fourth, implementation studies should measure pathway-level outcomes, including referral completion, diagnostic confirmation, treatment appropriateness, overtreatment, patient adherence, cost, and user acceptability (65, 67).

Data governance is also central to equitable deployment. Remote screening and mHealth systems may collect intraoral images, behavioral data, sensor-derived brushing metrics, salivary or systemic biomarker information, and longitudinal clinical histories. These data require clear consent processes, privacy protection, cybersecurity safeguards, audit trails, and policies for data storage, sharing, model updating, and secondary use. In addition, transparency should be tailored to different users: clinicians need information about model limitations, uncertainty, and appropriate use, patients need understandable explanations of what the tool can and cannot determine, and health systems need evidence on safety, cost, workflow impact, and liability.

Finally, equity safeguards must address implementation conditions, not only algorithmic performance. Tools intended to expand access may fail if they depend on expensive devices, stable internet connectivity, high digital literacy, or referral pathways that are unavailable to the populations being screened. Future studies should therefore evaluate affordability, device access, language and health-literacy adaptation, caregiver involvement, clinician training, reimbursement models, and post-deployment performance drift. In this sense, fairness should be treated as a lifecycle requirement (62, 64, 66). It begins with representative data and co-design, continues through external validation and subgroup auditing, and extends into monitoring after real-world deployment.

### 9.5 Limitations

This review has several limitations. The synthesis was narrative rather than systematic; PRISMA reporting guidelines were not applied, no PROSPERO registration was undertaken, and study selection was performed by a single reviewer without duplicate independent screening. Selection bias is therefore possible, particularly in the inclusion of pre-identified foundational references. The search was restricted primarily to PubMed-indexed literature published between January 2022 and April 2026, with selected earlier references retained when directly relevant to key concepts. Important studies indexed in non-MEDLINE databases, non-English publications, computer-science conference proceedings, regulatory documents, and grey literature may not have been captured.

Several conclusions also rely on systematic reviews and overviews of heterogeneous primary studies, so the synthesis is limited by the quality, reporting standards, and possible overlap of the underlying evidence base. Because the field is evolving rapidly, evidence published after the search cut-off is not reflected. The conclusions should therefore be interpreted as a structured narrative synthesis of the available evidence rather than a definitive or exhaustive assessment of oral-health AI.

## 10. Conclusions

AI is becoming increasingly capable of detecting and predicting selected oral-health conditions, particularly in image-based tasks such as caries detection, periodontal bone-loss assessment, and oral cancer or OPMD screening. However, the evidence reviewed here indicates that diagnostic performance alone is not sufficient to justify broad clinical implementation. Many studies still rely on single-center datasets, heterogeneous reference standards, incomplete demographic reporting, limited external validation, and outcomes that focus on accuracy rather than patient benefit.

The most clinically meaningful direction for oral-health AI is the shift from remote screening toward precision prevention. In this model, AI is not used only to classify lesions, but to combine imaging, behavioral data, salivary biomarkers, longitudinal risk profiles, and clinician support to guide prevention, referral, follow-up, and treatment decisions. Early evidence from AI-enabled mHealth and multimodal feedback systems suggests that these approaches may improve oral-hygiene behavior and periodontal inflammatory outcomes, but larger, more diverse, and longer-term, and implementation-focused studies are needed to determine durability, affordability, scalability, adherence and equity impact.

Responsible implementation will require external validation across populations, devices, and care settings; transparent reporting of dataset composition and subgroup performance; calibration and uncertainty assessment; fairness auditing; clinician training; privacy and data-governance safeguards; and post-deployment monitoring for model drift, safety, access, and equity impact. Future research should prioritize prospective pathway-level evaluations that measure not only diagnostic accuracy, but also referral completion, confirmatory diagnosis, treatment appropriateness, overtreatment, tooth retention, periodontal stability, cost-effectiveness, and patient experience. Equity must also be treated as a design requirement rather than a secondary consideration. Tools intended to expand access could reinforce disparities if they are unaffordable, poorly governed, trained on unrepresentative data, or disconnected from confirmatory diagnosis and care pathways. The central test for oral-health AI will therefore be whether it can improve prevention and access in the populations intended for use, rather than whether it can achieve higher accuracy in isolated datasets.

Overall, oral-health AI should currently be viewed as a promising decision-support and prevention-support technology, not as an autonomous substitute for dental professionals or public-health infrastructure. Its value will depend less on additional gains in isolated diagnostic accuracy and more on whether it can be validated, governed, and integrated into equitable care systems that improve prevention and outcomes for the populations most affected by oral disease.
