# Supplementary material for: From remote screening to precision prevention: responsible multimodal AI for risk prediction and equitable oral healthcare
Source: Front Oral Health. 2026 Jul 24;7:1878692. doi: 10.3389/froh.2026.1878692 (PMC13447499; doi:10.3389/froh.2026.1878692)
Supplement: Supplementary file 1 [file Table1.docx]

**Supplementary Table 1. Structured PubMed search strategy by thematic domain**

Approximate hits indicate domain-level PubMed yields at the time of searching. Records triaged indicate the approximate number of records screened for relevance within each thematic domain. Counts were not deduplicated across domains and should not be interpreted as a PRISMA-style flow record.

| **Domain** | **Search terms** |
| --- | --- |
| **Landscape and framing** | ("artificial intelligence"[MeSH] OR "deep learning"[MeSH] OR "machine learning"[MeSH]) AND ("dentistry"[MeSH] OR "oral health"[MeSH] OR dental[Title/Abstract]) AND (review[Publication Type] OR "scoping review"[Title/Abstract] OR "umbrella review"[Title/Abstract] OR "narrative review"[Title/Abstract] OR editorial[Publication Type])  PubMed results: 394 |
| **Caries detection** | ("artificial intelligence"[MeSH] OR "deep learning"[MeSH] OR "machine learning"[MeSH] OR "convolutional neural networks"[Title/Abstract]) AND ("dental caries"[MeSH] OR caries[Title/Abstract] OR "early childhood caries"[Title/Abstract]) AND (detection[Title/Abstract] OR diagnosis[Title/Abstract] OR prediction[Title/Abstract] OR screening[Title/Abstract])  PubMed results: 200 |
| **Periodontal disease** | ("artificial intelligence"[MeSH] OR "deep learning"[MeSH] OR "machine learning"[MeSH]) AND ("periodontitis"[MeSH] OR "periodontal diseases"[MeSH] OR "alveolar bone loss"[MeSH] OR periodontal[Title/Abstract]) AND (detection[Title/Abstract] OR diagnosis[Title/Abstract] OR screening[Title/Abstract] OR prediction[Title/Abstract] OR risk[Title/Abstract])  PubMed results: 256 |
| **Oral cancer and oral potentially malignant disorders** | ("artificial intelligence"[MeSH] OR "deep learning"[MeSH] OR "machine learning"[MeSH]) AND ("mouth neoplasms"[MeSH] OR "oral cancer"[Title/Abstract] OR "oral squamous cell carcinoma"[Title/Abstract] OR "potentially malignant disorder"[Title/Abstract] OR OPMD[Title/Abstract] OR "oral leukoplakia"[Title/Abstract]) AND (detection[Title/Abstract] OR screening[Title/Abstract] OR diagnosis[Title/Abstract] OR prediction[Title/Abstract])  PubMed results: 322 |
| **Multimodal AI and mHealth** | (("artificial intelligence"[MeSH] OR "deep learning"[MeSH]) AND ("mobile applications"[MeSH] OR "smartphone"[MeSH] OR mHealth[Title/Abstract] OR "mobile health"[Title/Abstract] OR multimodal[Title/Abstract])) AND ("oral health"[MeSH] OR dental[Title/Abstract] OR oral[Title/Abstract])  PubMed results: 119 |
| **Teledentistry, equity, and algorithmic fairness** | ("teledentistry"[Title/Abstract] OR "telemedicine"[MeSH]) AND ("oral health"[MeSH] OR dental[Title/Abstract]) AND (equity[Title/Abstract] OR access[Title/Abstract] OR underserved[Title/Abstract] OR rural[Title/Abstract] OR "low-income"[Title/Abstract] OR bias[Title/Abstract] OR sustainable[Title/Abstract] OR "health disparities"[Title/Abstract])  PubMed results: 169 |
